# Autoimmune psychosis: a review of diagnostic and treatment guidelines

**DOI:** 10.1192/j.eurpsy.2023.565

**Published:** 2023-07-19

**Authors:** P. Barbosa, M. Remelhe, L. Ribeiro

**Affiliations:** Psychiatry, Centro Hospitalar Vila nova de Gaia/Espinho, Vila Nova de Gaia, Portugal

## Abstract

**Introduction:**

Over this decade, there has been progressive growth and evolution of the concept of autoimmune encephalitis. However, international consensus overly focus on major neurological signs, while discarding some attenuated presentations, sometimes with just psychiatric manifestations. It was only very recently that a new concept arouse from this disorder, named autoimmune psychosis, which can mimic schizophrenia. Unfortunately, there is still a lack of a structured approach of psychotic patients to cover this disorder. This has numerous implications, namely on management and prognosis of these patients. Not only, these patients have an increased risk of neuroleptic malignant syndrome, but it is also important to intervene early in the course of disease.

**Objectives:**

To conduct a review of the diagnostic and treatment guideliens of autoimmune psychosis

**Methods:**

The authors conducted a non-systematic review, by resorting to the pubmed database, on the concept of autoimmune psychosis and updated proposals of diagnostic orienting lines.

**Results:**

Recently, in 2016, Graus et al proposed diagnostic criteria for possible autoimmune encephalitis, in which the authors acknowledge subacute onset of psychiatric symptoms as a possible clinical manifestation. The authors accept normal diagnostic tests, provided that new neurological focal findings exist. Since then, there have been described a list of signs/symptoms, which should raise suspicion for this diagnostic on psychiatric patients, so called red flags. In accordance to diagnostic guidelines for autoimmune psychosis, defined by Pollak et, the presence of this symptoms should lead clinicians to perform diagnostic exams, as MRI, eletroencephalogram and blood serological tests and lumbar puncture. However, others criticize this initial lineup arguing that some patients could be missed, because they do not have any neurological signs, and so they propose new diagnostic criteria.

**Conclusions:**

Autoimmune psychosis represents an attenuated clinical form of autoimmune encephalitis, although demanding the same medication and prompt initiation of treatment as other autoimmune encephalitis. It is important to acknowledge that there are patients who are seronegative and that some of the diagnostic exams mentioned have sometimes limited availability. As acknowledged by Guasp et al, there are patients with first psychotic episodes that have an autoimmune etiology, but because of the lack of neurological signs, could potentially be missed of treatment. So, it is important to establish formal diagnostic guidelines for this disorder, namely orienting lines for first psychotic episodes, which is the most commson psychiatric manifestation. This also enlightens the need for neurologic and psychiatric cooperation for these patients.

**Disclosure of Interest:**

None Declared

